# Expert consensus on an open‐access United Nations International Multiple Micronutrient Antenatal Preparation–multiple micronutrient supplement product specification

**DOI:** 10.1111/nyas.14322

**Published:** 2020-03-09

**Authors:** The Multiple Micronutrient Supplement Technical Advisory Group (MMS‐TAG), The Micronutrient Forum (MNF)

**Affiliations:** ^1^ Nutrition Science The New York Academy of Sciences New York City New York; ^2^ info@micronutrient.org

**Keywords:** product specification, multiple micronutrient supplement (MMS), UNIMMAP, packaging, manufacturing, quality, standards

## Abstract

The multiple micronutrient supplement (MMS) based on the United Nations International Multiple Micronutrient Antenatal Preparation (UNIMMAP) formula provides women and their offspring with a healthy start to life in an efficacious, safe, and cost‐effective way. To date, however, no precise and transparent specifications exist to support the manufacturing and distribution of UNIMMAP–MMS globally. To palliate for this need, the MMS Technical Advisory Group at the New York Academy of Sciences and the Micronutrient Forum convened a technical consultation to develop an open access UNIMMAP–MMS Product Specification for the manufacturing of this product. The specifications offered in this paper cover: ingredients, excipients, and processing aids used in the manufacturing of UNIMMAP–MMS; stability studies recommended under different testing conditions and climatic zones; packaging considerations; manufacturing standards, including pharmacopeia standards, manufacturing practices, certificates of analysis, change control, and quality agreement; finished product specifications, including tablet characterization and purity, potency assay; analytical test methods; and storage and transportation requirements.

## Graphical abstract

This paper results from deliberations of a panel of international experts assembled in Washington DC, November 11 and 12, 2019 and commissioned by the MMS Technical Advisory Group at the New York Academy of Sciences and the Micronutrient Forum to ensure consistency in the manufacturing of quality and affordable UNIMMAP–MMS across antenatal care programs, while allowing for variances in national regulatory and pharmacopoeia standards.

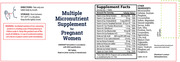

## Introduction

Micronutrient deficiencies can have deleterious impacts on maternal health and pregnancy outcomes.^1^ The United Nations International Multiple Micronutrient Antenatal Preparation–multiple micronutrient supplement (UNIMMAP–MMS) formulation^2^ has been shown to provide pregnant women and their offspring with a positive pregnancy and a healthy start to life in a safe^3^ and cost‐effective^4^ manner. The strength of the evidence, combined with global advocacy and the availability of technical assistance, drives an increased demand globally for the UNIMMAP–MMS product. The surge in demand, however, far exceeds global supply: most of the nearly 200 million pregnancies that occur annually in low‐ and middle‐income countries are likely to benefit from the use of MMS; yet, current production covers only about five millions of those pregnancies. Reaching an adequate level of supply will bring several new manufacturers into production. This paper, which results from the deliberations of a panel of international experts assembled in Washington DC, November 11–12, 2019, was commissioned by the MMS Technical Advisory Group at the New York Academy of Sciences and the Micronutrient Forum to ensure consistency in the manufacturing of quality and affordable UNIMMAP–MMS across antenatal care programs, while allowing for variances in national regulatory and pharmacopoeia standards. Further, the paper provides the basis for a quality agreement between a purchaser and a manufacturer by providing both parties with a clear technical understanding of the manufacturing requirements for the UNIMMAP–MMS product; and the means and methods to verify that the product delivered meets the quality that was expected by the purchaser.

## 1 Product description

The product defined by the following specification conforms to the UNIMMAP) formula and is an MMS for pregnant women that is delivered in the form of a film coated tablet.aThis specification states a preference for a film coated tablet, which provides certain benefits, including lower cost and better performance and stability under expected conditions of high temperature and humidity. However, capsules that demonstrate equal or better performance may also be considered.


## 2 Ingredients

### 2.1 Food/dietary/nutritional ingredients

Table 1 shows the food/dietary/nutritional ingredients used in the UNIMMAP formulation and should be prepared from ingredients that meet United States Pharmacopeia (USP) or other globally recognized pharmacopeia compendial standards.bReaders should consider the following: (1) UNIMMAP–MMS might be considered a medicinal product in some countries, and if so, the product must comply with the respective regulatory requirements of that country; (2) no reference is made in this document to “pharmaceutical” or “medicinal” ingredients or products, although the product might be considered a medicinal product in some countries; and (3) this specification outlines the minimum requirements for the manufacture of a UNIMMAP–MMS; as previously indicated, if a country has stricter requirements, they must be met. Where such standards do not exist, ingredients may be used in the UNIMMAP formulation if they have been shown to be of acceptable food grade quality using other suitable procedures.

**Table 1:** Recommended food/dietary/nutritional ingredients

Component	Chemical entity^a^	Amount
Vitamin A	Retinyl acetate	800 mcg RAE
Vitamin C	Ascorbic acid	70 mg
Vitamin D	Cholecalciferol	5 mcg (200 IU)
Vitamin E	Alpha tocopheryl succinate	10 mg α‐TE
Vitamin B1	Thiamine mononitrate	1.4 mg
Vitamin B2	Riboflavin	1.4 mg
Vitamin B3	Niacinamide	18 mg NE
Vitamin B6	Pyridoxine HCl	1.9 mg
Folic acid	Folic acid	680 mcg DFE (400 mcg)
Vitamin B12	Cyanocobalamin	2.6 mcg
Iron	Ferrous fumarate	30 mg
Iodine	Potassium iodide	150 mcg
Zinc	Zinc oxide	15 mg
Selenium	Sodium selenite	65 mcg
Copper	Cupric oxide	2 mg

a These chemical entities may be replaced by other chemical entities if they demonstrate equal or better performance (e.g., stability).

### 2.2 Excipients

Excipients used in the UNIMMAP formulation generally are prepared from ingredients that meet USP, National Formulary (NF), Food Chemical Codex, or other globally recognized pharmacopeia compendial standards. Where such standards do not exist, ingredients may be used in the UNIMMAP formulation if they have been shown to be of acceptable food grade quality using other suitable procedures.

Ingredients may be added to the UNIMMAP formulation provided that the ingredients comply with applicable regulatory requirements, and do not interfere with the assay and tests prescribed for determining compliance with the bulk or finished UNIMMAP–MMS product specification.

### 2.3 Processing aids or other materials

Processing aids or other materials used in the manufacture of the UNIMMAP formulation that do not end up in the finished product should be of acceptable food grade quality using suitable procedures.

Potable water must meet, at minimum, all the requirements for drinking water promulgated in the U.S. Environmental Protection Agency's National Primary Drinking Water Regulations (40 CFR Part 141), and any applicable state and local drinking water requirements that are more stringent. For manufacturers outside the United States, potable water meeting equivalent requirements may be acceptable with justification, for example, the drinking water regulations of the European Union (European Commission Directive 98/93/EC) or Japan Drinking Water Quality Standards. Water not meeting such requirements should not be permitted for use in the water purification system for *purified water*.

## 3 Stability studies

The UNIMMAP–MMS finished product labeling must state a shelf‐life (expiration) date that is indicative of the date before which the product is ensured to meet applicable specifications of identity, strength, quality, and purity when stored under labeled conditions. The shelf‐life (expiration) date must be supported by suitable stability data, following the guidelines in International Council for Harmonisation of Technical Requirements for Pharmaceuticals for Human Use (ICH) Q1A.

A documented ongoing testing program must be designed to monitor the stability characteristics of the UNIMMAP–MMS, and the results must be used to establish appropriate storage conditions and shelf‐life (expiration) dates for the UNIMMAP–MMS finished product. Test procedures used in stability testing must be validated and indicate stability. Stability samples should be stored in container‐closure systems that simulate the packaging proposed to distribute the finished UNIMMAP–MMS for consumer/patient use. Stability studies should include testing of those attributes of the dietary supplement that are susceptible to change during storage and that influence the quality of the dietary supplement.

The first three production batch(es) should be placed on the stability monitoring program to establish the product shelf‐life (expiration) date. The lots should be those that are manufactured at the regular manufacturing scale; however, two of the three production batches can be at least 1/10th the size of the manufacturing scale. Thereafter, at least one batch per year of manufactured UNIMMAP–MMS should be added to the stability monitoring program. All batches must comply with the finished product specification throughout the product shelf‐life (expiration) date.

As appropriate, the stability storage conditions for temperature and relative humidity for product intended for use globally should be for Climate Zone IVb, hot and very humid. However, actual climatic conditions in the country of destination of the UNIMMAP–MMS may require stability studies to be carried out under the conditions of a different climatic zone (e.g., Climatic Zone III, hot and dry). Table 2 shows the recommended testing conditions appropriate to each climatic zone that might be required in a given country of destination of the product.

**Table 2:** Recommended International Conference on Harmonization Testing conditions for all climatic zones

Climatic zone	Climate definition	Mean temperature/mean partial water vapor pressure	Derived climatic conditions	Long‐term stability	Accelerated stability
I	Temperate	NMT 15 °C/LT 11 hPa	21 °C/45% RH	25 °C ± 2 °C/60% RH ± 5% RH	40 °C ± 2 °C/75% RH ± 5% RH
II	Subtropical, Mediterranean	GT 15 °C and NMT 22 °C/GT 11 hPa and NMT 18 hPa	25 °C/60% RH	25 °C ± 2 °C/60% RH ± 5% RH	40 °C ± 2 °C/75% RH ± 5% RH
III	Hot, dry	GT 22 °C/LT 15 hPa	30 °C/35% RH	30 °C ± 2 °C/35% RH ± 5% RH	40 °C ± 2 °C/NMT 25%
IVa	Hot, humid	GT 22 °C/GT 15 hPa and NMT 27 hPa	30 °C/65% RH	30 °C ± 2 °C/65% RH ± 5% RH	40 °C ± 2 °C/75% RH ± 5% RH
IVb	Hot and very humid	GT 22 °C/GT 27 hPa	30 °C/75% RH	30 °C ± 2 °C/75% RH ± 5% RH	40 °C ± 2 °C/75% RH ± 5% RH

NMT, not more than (≤); LT, less than (<); GT, greater than (>); RH, relative humidity.

The frequency of testing should be sufficient to establish the stability profile of the dietary supplement. The storage conditions and length of studies chosen should be sufficient to cover storage, shipment, and subsequent use. Data from the accelerated storage condition can be used to evaluate the effect of short‐term excursions outside the label storage conditions, such as might occur during shipping. The shelf‐life (expiration) period of the UNIMMAP–MMS should be 30 months, at minimum. The following testing frequencies are recommended:

- Long term = 0, 3, 6, 9, 12, 18, 24, 30, and 36 months;
- Accelerated = 0, 3, and 6 months.

Where an expectation (based on development experience) exists that results from accelerated studies are likely to approach significant change criteria, increased testing should be conducted by adding samples at the 1‐ and 2‐month time points. In general, a significant change for a dietary supplement is defined as a 5% change in the assay from its initial value; or failure to meet any of its product specification acceptance criteria.

## 4 Packaging

### 4.1 Package types and tablet count

This specification focuses on the use of UNIMMAP–MMS packaged in bottles containing 180 tablets per bottle. Bottles (or blister packaging) containing 30 tablets are acceptable, but packaging options/tablet counts should be considered in light of cost and environmental implications, and evidence that may come later as to the impact that a particular packaging option or tablet count may have on distribution, uptake, adherence rates, or clinic attendance.cNo evidence currently exists yet in the literature indicating that packaging type/tablet count increases uptake, adherence rates, or clinical attendance. However, the 180‐count bottled product makes it less expensive per tablet to dispense and has a lesser environmental impact than other packaging. For these reasons, the 180‐count‐HDPE‐bottled product was selected as the packaging option of choice in the preparation of this document. If future implementation research, or if program needs indicate otherwise, other packaging options may be considered with appropriate modifications to these technical specifications.


Bulk packaging for business‐to‐business transactions is acceptable with demonstrated stability. Bulk packaging for clinics (e.g., 500, 1000, etc., count bottles) should be avoided due to health and safety concerns to avoid inadvertent child exposure to UNIMMAP–MMS; and to avoid repackaging by clinic staff of MMS into other temporary less desirable packaging (e.g., plastic bags and newspaper), which may cause premature product deterioration.

### 4.2 Bottles

Bottles must be:

- White/opaque
- Screw cap
- High‐density polypropylene (HDPE) material (complying with internationally recognized pharmacopoeia standards)
- Tamper evident
- Child resistant

The need for desiccant depends on tablet formulation and must be determined by the manufacturer based on experience and supporting stability data.

### 4.3 Blister packaging

If blister packaging is used, a thermoformable moisture barrier film, such as Aclar^®^, should be used to ensure the stability of the product throughout the shelf‐life of the product. Using a child‐resistant version of a blister pack is deemed impractical, as the “child‐resistant” requirement makes a blister pack unusually difficult to dispense package contents.

### 4.4 Labeling

Labeling must comply with applicable country of destination regulatory requirements for the food/dietary/nutritional supplement.

Quantitative label claims for the product must be truthful and accurately reflect the contents of the declared food/dietary/nutritional ingredients; fortified or fabricated nutrients must meet 100% of the quantitative label claim throughout the shelf‐life of the product for products that can be distributed in the United States. For products distributed outside the United States, fortified or fabricated nutrients must meet USP compendial assay acceptance criteria (see Tables 3 and 4 for details).

**Figure 1:**
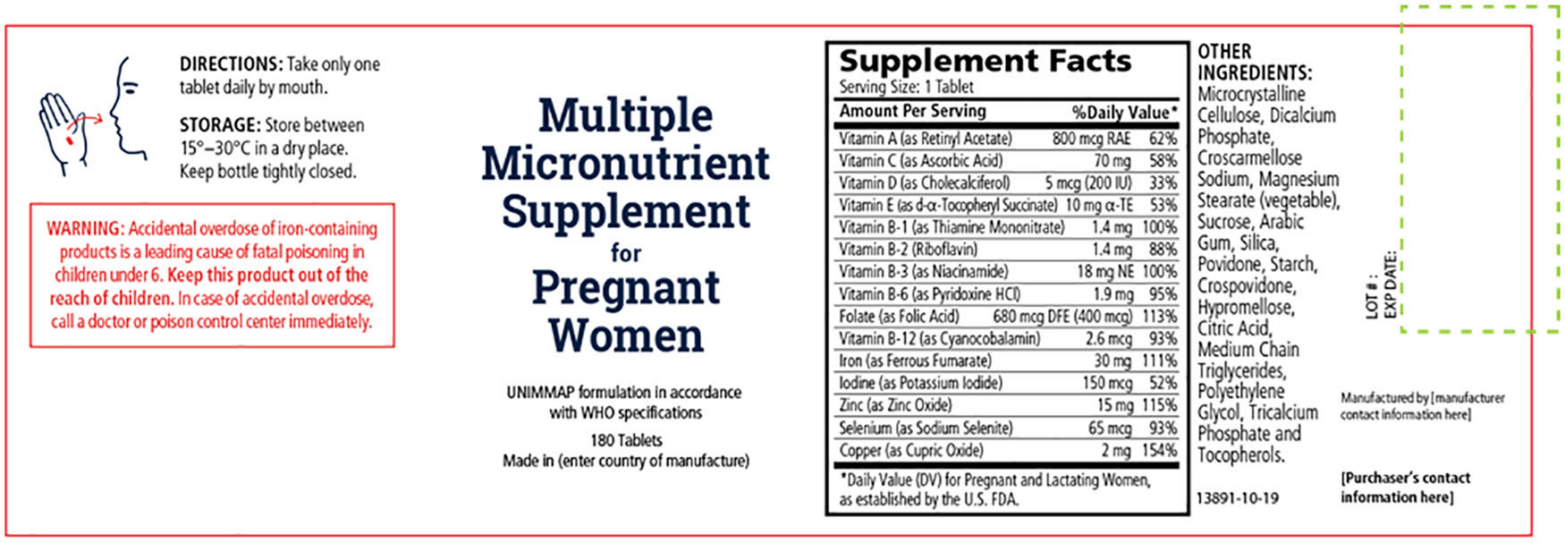
Label illustrating the minimum recommended information that should appear on the label of the UNIMMAP–MMS.

The UNIMMAP–MMS product label (see Fig. 1) should list:

- The term “food supplement,” “dietary supplement,” or “nutritional supplement”;
- The quantity of each dietary ingredient and the correct reference daily intake, listed as % Daily Value, as necessary;
- List the common or usual name of each ingredient in descending order of predominance by weight, except that dietary ingredients listed in the nutrition label supplement facts need not be repeated in the ingredient list; incidental additives, including water, present in a dietary supplement at insignificant levels are exempted from this requirement.

Labels for the UNIMMAP–MMS containing iron or iron salts for use as an iron source must include the following required cautionary statement: “WARNING: Accidental overdose of iron‐containing products is a leading cause of fatal poisoning in children under 6‐years of age. Keep this product out of reach of children. In case of accidental overdose, call a doctor or poison control center immediately.”

The label must include a statement of the necessary storage requirements for the UNIMMAP–MMS. The label must accurately state the country of origin for any product of foreign origin imported into the country of destination.

The name and place of business of the manufacturer, packer, or distributor, and the expiration date must be located to the right of the principle display panel. When the name appearing on the label is not that of the actual manufacturer, the name should be qualified in a manner to accurately reflect this relationship (e.g., “Manufactured for ___,” “Distributed by ___”). The label must include a domestic address or phone number through which an adverse event report for a dietary supplement may be received.

## 5 Manufacturing standards and certificates

### 5.1 Pharmacopoeia standards

Compliance to the following international pharmacopoeial standards is acceptable for food/dietary/nutritional supplement ingredients and finished product:

- United States Pharmacopoeia (USP);
- European Pharmacopoeia (Ph.Eur.);
- International Pharmacopoeia (Ph.Int.);
- British Pharmacopoeia (BP);
- Japanese Pharmacopoeia (JP).

### 5.2 Manufacturing practices and conditions

The UNIMMAP–MMS must be manufactured under current good manufacturing practice (cGMP) regulations promulgated by an internationally recognized regulatory authority (e.g., U.S. FDA and MHRA), or other GMP guidelines by a stringent authority (e.g., World Health Organization (WHO)), by a PIC/S member, or by a globally recognized pharmacopeia (e.g., USP), including but not limited to:

- U.S. FDA 21 CFR Part 111 Current Good Manufacturing Practice in Manufacturing, Packing, Labeling, or Holding Operations for Dietary Supplements; and
- USP–NF general chapter 〈2750〉 Manufacturing Practices for Dietary Supplements.

The manufacturing site and operations should be audited by an accredited third‐party certification body.

### 5.3 Certificates of analysis

Certificates of analysis (CoA) must be issued for each batch of UNIMMAP–MMS product. The CoA should list the name and item code of the UNIMMAP–MMS product, batch number, tested and released dates, and the expiration date. The CoA should list each test performed, the specific identity of the test procedure, the acceptance limits or criteria, and the results with numerical units, as appropriate. The CoA should be dated and signed by authorized quality unit personnel. For CoAs issued for external use, the CoA should list the name, address, and telephone number of the manufacturer.

The testing protocol should include the performance of full specification testing. However, a reduced level of testing (or sampling) for particular specified parameters may be allowed based upon one or more of the following: statistical analysis of an adequate quantity of historical test data; statistical confidence in the capability of the manufacturing process as determined by suitable verification; or ongoing monitoring of the process using recognized statistical process control techniques. The manufacturer must notify the purchaser of the UNIMMAP–MMS product in writing of any change to the testing protocol.

### 5.4 Halal certification

The UNIMMAP–MMS product may be manufactured meeting Halal requirements. The exact requirements shall be obtained from local authorities or from an accredited source.

### 5.5 Change control

A manufacturer might make changes to the UNIMMAP–MMS product's specification, raw material source, manufacturing processing steps and/or equipment, testing protocols, or any other criteria deemed to be essential or significant to product quality. Manufacturers must notify the purchaser of the UNIMMAP–MMS product of any significant changes that might affect product quality, in writing upon implementation, along with the rationale for the change(s).

A manufacturer must also notify the purchaser of the UNIMMAP–MMS product of any change to its manufacturing site, including any changes to the certification status held by the manufacturer from a GMP issuing authority.

### 5.6 Quality agreement

There should be a written and approved contract or quality agreement between the contract giver and the contract acceptor that defines in detail the GMP responsibilities, including the quality measures, of each party. The contract should permit the purchaser to audit the manufacturer's (seller's) facilities for compliance with GMPs. Where subcontracting is allowed, the seller should not pass to a third party any of the work entrusted to it under the contract without the buyer's prior evaluation and approval of the arrangements.

## 6 Finished product specification

The UNIMMAP is a formulation for use by pregnant women. UNIMMAP–MMS was developed during a workshop of experts organized by the WHO, UNICEF, and the United Nations University in 1999 specifically to identify an MMS formula for efficacy clinical trials. It contains 15 micronutrients at dosages that approximate the recommended dietary allowances for pregnancy.

Tables 3 and 4 show criteria and requirements for tablet characterization and purity, as well as the potency assay requirements.

**Table 3:** Tablet characterization and purity

Test	Test method	Acceptance criteria
Physical characteristics		
Appearance	Visual	TBD by manufacturer
Shape	Visual	TBD by manufacturer
Tablet thickness	Micrometer	TBD by manufacturer
Tablet length	Micrometer	TBD by manufacturer
Tablet friability	USP <1216>	TBD by manufacturer
Tablet breaking force	USP <1217>	TBD by manufacturer
Performance		
Average tablet weight	USP <2091>	TBD by manufacturer
Weight variation		Each of the individual weights is within 95–105% of the average weight
Dissolution for vitamin A (index for oil‐soluble vitamins)	USP <2040> Apparatus 2, at 75 rpm, in 0.05 M phosphate buffer pH 6.8, w/1% (w/v) sodium ascorbate and 1% (w/v) octoxynol 9, 900 mL	LT 75% of the labeled amount of vitamin A dissolved in 45 minutes
Dissolution for folic acid	USP <2040> Apparatus 2, at 75 rpm, in water or 0.05 M pH 6.0 citrate buffer, 900 mL	LT 75% of the labeled amount of folic acid dissolved in 1 hour
Dissolution for riboflavin (index for water‐soluble vitamin)	USP <2040> Apparatus 2, at 75 rpm, in 0.1 N hydrochloric acid, 900 mL	LT 75% of the labeled amount of riboflavin dissolved in 1 hour
Dissolution for iron (index element)		LT 75% of the labeled amount of iron dissolved in 1 hour
Elemental impurities		
Arsenic (inorganic)	USP <233> and USP <2232>	NMT 15 mcg/day
Cadmium		NMT 5 mcg/day
Lead		NMT 5 mcg/day
Mercury (total)		NMT 15 mcg/day
Methylmercury (as Hg)^e^		NMT 2 mcg/day
Microbial contaminants		
Total aerobic microbial count (TAMC)	USP <2021>	NMT 3 × 10^3^ CFU/g
Total combined yeast and mold (TCYM)	USP <2021>	NMT 3 × 10^2^ CFU/g
Absence of *Escherichia coli*	USP <2022>	Absent in 10 g
Absence of *Salmonella* spp.	USP <2022>	Absent in 10 g
Absence of *Staphylococcus aureus*	USP <2022>	Absent in 10 g
Enterobacterial count (bile‐tolerant Gram‐negative bacteria)	USP <2021>	NMT 10 CFU/g

NMT, not more than (≦); LT, less than (<); CFU, colony forming unit.

USP <233> Elemental Impurities‐Procedures.

USP <2232> Elemental Contaminants in Dietary Supplements.

USP <2021> Microbial Enumeration Tests – Nutritional and Dietary Supplements.

USP <2022> Microbiological Procedures for Absence of Specified Microorganisms – Nutritional and Dietary Supplements.

**Table 4:** Potency assay (per tablet)

Ingredient	Test method^a^	Label claim	USP	US‐FDA^b^
Vitamin A (as retinyl acetate)	Vitamin A, method 1	800 mcg	LT 90.0% NMT 165.0%	LT 100.0% NMT 175.0%
Vitamin C (as ascorbic acid)	Vitamin C, assay <580>, method II	70 mg	LT 90.0% NMT 150.0%	LT 100.0% NMT 160.0%
Vitamin D (as cholecalciferol)	Cholecalciferol or ergocalciferol method 1	5 mcg (200 IU)	LT 90.0% NMT 165.0%	LT 100.0% NMT 175.0%
Vitamin E (as dl‐alpha tocopheryl succinate)	Vitamin E, method 1	10 mg	LT 90.0% NMT 165.0%	LT 100.0% NMT 175.0%
Vitamin B1: thiamine (as thiamine mononitrate)	Niacin or niacinamide, pyridoxine hydrochloride, riboflavin, and thiamine; method 1	1.4 mg	LT 90.0% NMT 150.0%	LT 100.0% NMT 160.0%
Vitamin B2: riboflavin		1.4 mg	LT 90.0% NMT 150.0%	LT 100.0% NMT 160.0%
Vitamin B3: niacin (as niacinamide)		18 mg	LT 90.0% NMT 150.0%	LT 100.0% NMT 160.0%
Vitamin B6: pyridoxine hydrochloride		1.9 mg	LT 90.0% NMT 150.0%	LT 100.0% NMT 160.0%
Folate (as folic acid)	Folic acid, method 1	680 DFE (400 mcg folic acid)	LT 90.0% NMT 150.0%	LT 100.0% NMT 160.0%
Vitamin B12 (as cyanocobalamin)	Cyanocobalamin, method 1	2.6 mcg	LT 90.0% NMT 150.0%	LT 100.0% NMT 160.0%
Iodine (as potassium iodide)	Iodide	150 mcg	LT 90.0% NMT 160.0%	LT 100.0% NMT 170.0%
Iron (as ferrous fumarate)	Copper, iron, and zinc, method 2; selenium, method 3 plasma spectrochemistry <730>	30 mg	LT 90.0% NMT 125.0%	LT 100.0% NMT 135.0%
Zinc (as zinc oxide)		15 mg	LT 90.0% NMT 125.0%	LT 100.0% NMT 135.0%
Selenium (as sodium selenite)		65 mcg	LT 90.0% NMT 160.0%	LT 100.0% NMT 170.0%
Copper (as cupric oxide)		2 mg	LT 90.0% NMT 125.0%	LT 100.0% NMT 135.0%

NMT, not more than (≦); LT, less than (<).

a All tests to be performed per current USP‐NF, oil‐ and water‐soluble vitamins with minerals tablets.

b USP General Notices 4.10.20. Acceptance Criteria: An official product shall be formulated with the intent to provide 100% of the quantity of each ingredient declared on the label. Where the minimum amount of a substance present in a dietary supplement is required by law to be higher than the lower acceptance criterion allowed for in the monograph, as per 21 CFR 101.36(f)(1) and 21 CFR 101.9(g)(3) and (g)(4), the upper acceptance criterion in the monograph may be increased by a corresponding amount.

## 7 Analytical test methods

Tests and examinations that are used to determine whether UNIMMAP–MMS specifications are met must be appropriate for their intended use, and scientifically validated methods. Test methods or procedures must meet proper standards of accuracy and reliability.

If the test procedure is not in an official compendium, the procedure must be validated according to USP general chapter <1225> Validation of Compendial Procedures or ICH Q2(R1) Validation of Analytical Procedures: Text and Methodology. Method performance characteristics include specificity, linearity, range accuracy, precision, detection limit, and quantitation limit, and those of interest may vary depending on the type of test: identification, assay, impurities, or performance.

If the test procedure is in an official compendium, such as USP‐NF, the procedure only needs to be verified for its suitability under actual conditions of use, according to USP general chapter <1226> Verification of Compendial Procedures. Verification requirements should be based on an assessment of the complexity of both the procedure and material to which the procedure is applied. Verification is not required for basic compendial procedures, such as loss on drying, residue on ignition, and simple instrumental determinations, such as pH measurements.

An alternative method or procedure is defined as any method or procedure other than the compendial method or procedure for the article in question. The alternative method or procedure must be fully validated and must produce comparable results to the compendial method or procedure within allowable limits established on a case‐by‐case basis. Alternative methods or procedures can be developed for any number of reasons not limited to simplification of sample preparation, enhanced precision and accuracy, improved (shortened) run time, or being better suited to automation than the compendial method or procedure. Only those results obtained by the methods and procedures given in the compendia are conclusive.

## 8 Storage and transportation requirements

The UNIMMAP–MMS product must be held under appropriate conditions of temperature, humidity, and light so that its identity, purity, strength, and composition are not affected (e.g., NLT 15 °C and NMT 30 °C, protected from humidity and light).

The UNIMMAP–MMS product must be distributed under conditions that will protect it against contamination and deterioration. The manufacturer and purchaser need to work together to ensure that this requirement is met.

All transportation operation must be conducted under such conditions and controls necessary to prevent UNIMMAP–MMS product from becoming adulterated during transportation. Responsibility for ensuring that transportation operations are carried out adequately must be assigned to competent supervisory personnel. Shippers, receivers, loaders, and carriers engaged in transportation must conduct all transportation operations under such conditions and controls necessary to protect the UNNIMAP‐MMS product from becoming adulterated during transportation. Such operations include, but are not limited to, taking effective measures such as:

- Segregation, isolation, or the use of packaging to protect the UNNIMAP‐MMS product from contamination from other articles in the same load;
- Use of vehicles and transportation equipment that are adequately designed and maintained in a sanitary condition to prevent the UNIMMAP–MMS product from becoming contaminated during transportation operations; and
- Use of vehicles and transportation equipment that are adequately designed, maintained, and equipped to transport UNNIMAP‐MMS product under adequate temperature and humidity control to prevent UNNIMAP‐MMS product from becoming adulterated during transportation.

The first manufactured batch of the UNIMMAP–MMS product should be distributed first. Distributing operations must be designed to facilitate its recall, if necessary.

## 9 Definitions and acronyms (Box 1)

**Box 1:** Definitions and acronyms

Term/abbreviation	Definition/long form
Accredited third‐party certification body	An accredited third‐party certification body means a third‐party certification body that meets the applicable requirements of ISO/IEC 17020:2012 and/or ISO/IEC 17065:2012 and is accredited to conduct audits or inspections according to the applicable standard or regulatory requirements.
Article	Article includes substances (such as excipients, food/dietary/nutritional ingredients, and in‐process material), products (such as food/dietary/nutritional supplements), and materials (such as packaging containers and closures, and labels).
Batch	Batch is a specific quantity of a food/dietary/nutritional supplement or other article that is intended to be uniform; that is intended to meet specifications for identity, purity, strength, and composition; and that is produced during a specified time period according to a single manufacturing record during the same cycle of manufacture.
Composition	Composition is the specified mix of food/dietary/nutritional ingredients and excipients in a food/dietary/nutritional supplement.
Code of Federal Regulations (CFR)	The CFR annual edition is the codification of the general and permanent rules and regulations published in the Federal Register by the executive departments and agencies of the Federal Government of the United States. It is structured into 50 subject matter titles; title 21 applies to food and drugs. Titles are broken down into parts, subparts, sections, and paragraphs.
Certificate of analysis (CoA)	CoA is a document relating specifically to the results of testing a representative sample drawn from a batch of material. The CoA should list each test performed in accordance with compendial or manufacturer requirements, including reference to the test procedure, acceptance limits, and the results obtained.
Country of destination	The country in which the product is intended to be marketed/used.
Excipients	Excipients are substances other than food/dietary ingredients that have been appropriately evaluated for safety and are intentionally included in a food/dietary supplement to do one or more of the following: aid in the manufacture of a food/dietary supplement; protect, support, or enhance stability, bioavailability, or user acceptability; assist in product identification; and/or enhance any other attribute of the overall safety or delivery of the food/dietary supplement during storage or use. The term excipient is sometimes used synonymously with the term inactive ingredients and other ingredients.
Food/dietary/nutritional ingredient	Food/dietary/nutritional ingredients are ingredients with an established nutritional value, namely, vitamins and minerals in their respective chemical entity.
Food/dietary/nutritional supplement ingredient	Food/dietary supplement ingredient includes food/dietary ingredients and excipients.
Food/dietary/nutritional supplement	Food/dietary/nutritional supplement is a product intended to supplement the diet that contains one or more food/dietary/nutritional ingredients, that is intended for ingestion in a tablet, capsule, or liquid form, that is not represented for use as a conventional food or as the sole item of a meal or the diet, and is labeled as a food/dietary/nutritional supplement, and that sometimes referred to as a multi‐micronutrient supplement (MMS).
Globally recognized pharmacopeia compendial standard	The following international pharmacopoeia's official compendial standards are considered globally recognized: 1. British Pharmacopoeia (BP) 2. European Pharmacopoeia (Ph.Eur.) 3. International Pharmacopoeia (Ph.Int.) 4. Japanese Pharmacopoeia (JP) 5. United States Pharmacopeia (USP)
International Conference on Harmonization (ICH)	The International Conference on Harmonization (ICH) of Technical Requirements for Registration of Pharmaceuticals for Human Use brings together regulatory authorities and pharmaceutical industry to discuss scientific and technical aspects of drug registration.
Micronutrient Forum (MN Forum)	The Micronutrient Forum serves as a global catalyst and convener for sharing expertise, insights, and experience relevant to micronutrients in all aspects of health promotion and disease prevention, with special emphasis on the integration with relevant sectors.
Not less than (NLT)	NLT is equal to, but not less than a given value.
Not more than (NMT)	NMT is equal to, but not more than a given value.
Pharmaceutical Inspection Co‐operation Scheme (PIC/S)	PIC/S is a nonbinding, informal co‐operative arrangement between regulatory authorities in the field of good manufacturing practice (GMP) of medicinal products for human or veterinary use. PIC/S currently consists of 52 participating authorities and aims at harmonizing inspection procedures.
To be determined (TBD)	TBD is related to a variable that has not yet been determined.
United Nations International Multiple Micronutrient Antenatal Preparation (UNIMMAP)	UNIMMAP is a formulation for a prenatal micronutrient supplement intended for use in developing countries that was developed in 1999 by UNICEF, the United Nations University (UNU), and the World Health Organization (WHO). It contains 15 micronutrients at dosages that approximate the recommended dietary allowances for pregnancy.
Vitamin Angel Alliance (VAA)	The Vitamin Angel Alliance, Inc. is a 501(c)(3) tax exempt organization that aims to reduce health and economic disparities across the life span of underserved populations by effectively delivering evidence‐based nutrition interventions to hard‐to‐reach populations globally (www.vitaminangels.org).

## Competing interests

The authors declare no competing interests.
